# Neuropilin-1 Regulates the Secondary CD8 T Cell Response to Virus Infection

**DOI:** 10.1128/mSphere.00221-19

**Published:** 2019-05-22

**Authors:** Ji Young Hwang, Yanbo Sun, Christopher R. Carroll, Edward J. Usherwood

**Affiliations:** a Department of Microbiology and Immunology, Geisel School of Medicine at Dartmouth College, Lebanon, New Hampshire, USA; University of Kentucky

**Keywords:** Kaposi’s sarcoma-associated herpesvirus, T cells, immune memory

## Abstract

Neuropilin-1 (Nrp1) plays important roles in axonal guidance in neurons and in the growth of new blood vessels. There is also a growing appreciation for roles played by neuropilin-1 in the immune response. This molecule is important for the function of regulatory T cells; however, roles in other T cell populations have not been identified. Here, we show that neuropilin-1 is expressed during the peak of the antiviral CD8 T cell response during murine gammaherpesvirus infection. Using a conditional knockout model, we deleted Nrp1 either before infection or after CD8 T cell memory had been established. We found that deletion of Nrp1 skewed the acute CD8 T cell response toward a memory precursor-like phenotype; however, the ensuing resting memory response was similar regardless of Nrp1 expression. Interestingly, Nrp1 deletion had differing effects on the recall response depending on the timing of deletion. When deleted before infection, Nrp1 deficiency inhibited the secondary response. Deletion just prior to reexposure to virus led to an enhanced secondary response. Interestingly, these effects were observed only in mice infected with a persistent strain of murine gammaherpesvirus and not with a nonpersistent mutant strain. These data highlight a multifaceted role for neuropilin-1 in memory CD8 T cell differentiation, dependent upon the stage of the T cell response and characteristics of the infectious agent. Several therapeutic anticancer therapies focus on inhibition of Nrp1 to restrict tumor growth, and so knowledge of how Nrp1 blockade may affect the CD8 T cell response will provide a better understanding of treatment consequences.

**IMPORTANCE** CD8 T cell responses are critical to control both virus infections and tumors. The ability of these cells to persist for long periods of time can result in lifelong immunity, as relatively small populations of cells can expand rapidly to counter reexposure to the same insult. Understanding the molecules necessary for this rapid secondary expansion is critical if we are to develop therapies that can provide lifelong protection. This report shows an important and complex role for the molecule neuropilin-1 in the secondary response. Several cancer therapies targeting neuropilin-1 are in development, and this work will lead to better understanding of the effect these therapies could have upon the protective CD8 T cell response.

## Abstract (precis)

CD8 T cell responses are critical to control both virus infections and tumors. The ability of these cells to persist for long periods of time can result in lifelong immunity, as relatively small populations of cells can expand rapidly to counter reexposure to the same insult. Understanding the molecules necessary for this rapid secondary expansion is critical if we are to develop therapies that can provide lifelong protection. This report shows an important and complex role for the molecule neuropilin-1 in the secondary response. Several cancer therapies targeting neuropilin-1 are in development, and this work will lead to better understanding of the effect these therapies could have upon the protective CD8 T cell response.

## INTRODUCTION

Neuropilin-1 (Nrp1) is a type I transmembrane protein with multiple domains that functions as a coreceptor for several ligands, such as semaphorins (SEMA), vascular endothelial growth factor (VEGF), and transforming growth factor beta (TGFβ). The molecule itself lacks kinase activity, but it associates with other receptors, such as integrins, plexins, and the VEGF receptor, that mediate transmembrane signaling (1). It was first studied in the nervous system, where neuropilin-1 is known to participate in neuronal development and provide cues for axonal guidance (2). Later, the interaction between VEGF and Nrp1 was found to play an important role in angiogenesis (3, 4). The involvement of Nrp1 in the growth of new blood vessels in tumor vasculature promotes tumor progression, and its blockade can restrict tumor growth (5, 6). Nrp1 can also be expressed by tumor cells themselves, and a peptide which inhibits VEGF-Nrp1 interactions has been shown to induce apoptosis of Nrp1-expressing breast tumor cells (7).

Tumors often elaborate an immunosuppressive microenvironment, and neuropilin-1 has been shown to play important roles in suppression mediated by regulatory T cells (Tregs). A recent study showed Nrp1 on Tregs was required for the suppression of the antitumor T cell response and to cure inflammatory colitis (8). Engagement of Nrp1 promoted Treg quiescence and limited differentiation, resulting in enhanced Treg stability in the tumor (8). The expression of Nrp1 differentiates natural from inducible regulatory T cells in some physiological settings (9, 10) and also identifies CD4^+^ CD25^−^ T cells with inhibitory function and the ability to recruit conventional Tregs (11). Interestingly, a recent report showed Nrp1 expression on group 3 innate lymphoid cells (ILC3s) in the lung with lymphoid tissue inducer activity and suggested functions in the early development of tertiary lymphoid aggregates in the lung and/or pulmonary angiogenesis (12). Collectively, these studies imply Nrp1 not only has a major impact in modulating responses to tumors but also plays a role in immune regulation and tissue remodeling in other physiological settings.

Nrp1 clearly plays important roles in immune regulatory cell populations; however, its role in conventional T cell populations has not been determined. CD8 T cells are important for controlling virus infections and restricting the growth of tumors, providing lifelong immunity by developing into memory cells that can respond rapidly to reinfection. Memory cells develop from memory precursors present early in the T cell response (13), and signals from cytokines, costimulatory molecules, and CD4 T cells are necessary for them to develop optimal recall responses (14). In this study, we investigated the role of Nrp1 on the CD8 T cell response to murine gammaherpesvirus (MHV-68) infection using a conditional knockout model. By means of this strain, we were able to restrict the deletion to CD8 T cells and regulate the timing of the deletion by tamoxifen administration.

We show that Nrp1 is highly upregulated on CD8 T cells during the acute phase of viral infection and that deletion of Nrp1 during this window skewed the T cells more toward memory precursors than terminally differentiated effector cells. Interestingly, “early” Nrp1 deletion resulted in weaker CD8 T cell expansion following virus rechallenge, suggesting Nrp1 signaling during priming promotes optimal “programming” of memory CD8 T cells. Interestingly, when deletion of Nrp1 occurred just before the recall response, the magnitude of the response was higher, indicating Nrp1 signals restrain the recall response. Interestingly, these effects were only observed with a persistent strain of MHV-68, but Nrp1 did not appear to affect recall responses in infection with a nonpersistent strain of the virus. What emerges is a complex role for Nrp1 in the CD8 T cell response, which is dependent upon both the timing of Nrp1 expression during the primary versus secondary response and the nature of the infection.

## RESULTS

### Nrp1 is upregulated with both persistent and nonpersistent MHV-68 infections.

**FIG 1:**
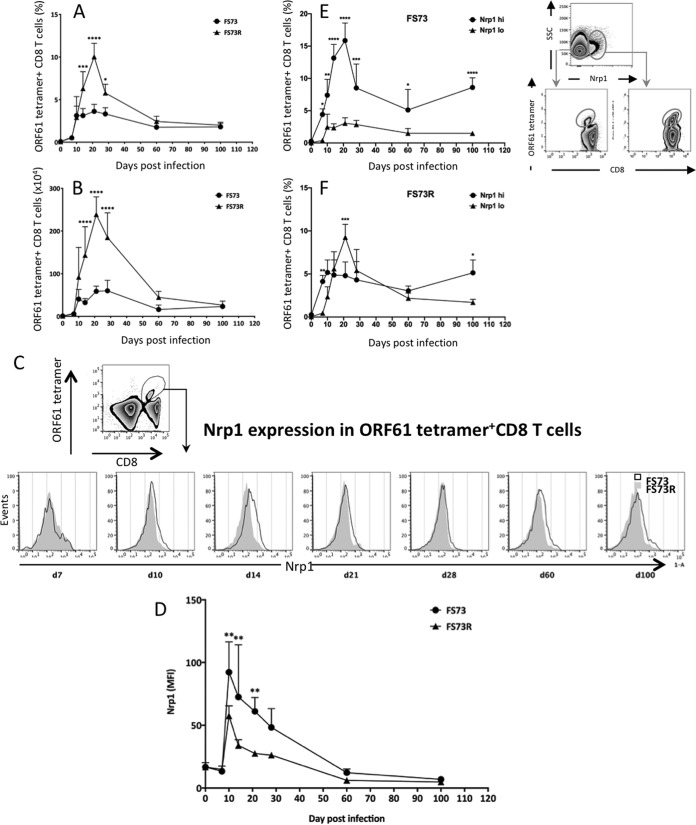
Nrp1 expression on CD8 T cells after persistent (FS73R) and nonpersistent (FS73) MHV-68 infection. (A) The proportions of ORF61-specific T cells among total splenic CD8 T cells after infection with either the FS73 or FS73R strain of MHV-68. (B) Numbers of ORF61-specific CD8 T cells in spleens of mice infected with either the FS73 or FS73R strain of MHV-68. (C) Histograms showing Nrp1 expression gated on CD8^+^ ORF61 tetramer^+^ splenocytes at the times postinfection shown. *y* axes in bottom plots are normalized to the mode. (D) Nrp1 mean fluorescence intensity (MFI) of tetramer^+^ CD8 T cells compared over time for FS73 and FS73R infection. Frequencies of ORF61 tetramer^+^ CD8 T cells that were Nrp1^−^ or Nrp1^+^ after FS73 infection (E, panels on right show representative plots showing gating strategy to distinguish Nrp1^hi^ and Nrp1^lo^ cells) or FS73R infection (F). All data show means ± standard deviations (SDs) from 4 to 5 mice per group. *, *P* < 0.05; **, *P* < 0.01; ***, *P* < 0.001; ****, *P* < 0.0001. Representative data from at least two experiments are shown.

Our previous research has shown that the CD8 T cell response differs between infection with a mutant MHV-68 with a deletion in ORF73 (FS73) (15, 16), which is essential for latent infection, and infection with a revertant virus that retains the ability to persist in the host. The original report of this mutant showed normal virus replication both *in vitro* and in the lungs of mice but the absence of latent infection, measured by either infectious center assay, *in situ* hybridization, or PCR (15). Studies from our own laboratory confirmed the absence of latency by real-time PCR, in addition to the absence of latency-associated splenomegaly and mononucleosis, and showed robust primary CD8 T cell responses induced by both FS73 and revertant viruses. However, the memory CD8 T cell phenotype differed, with higher turnover, lower Bcl-2 expression, and lower IL-2 expression during the persistent infection (16). To understand the role of Nrp1 on CD8 T cells upon MHV-68 infection, we initially measured the kinetics of Nrp1 expression on CD8 T cells after either persistent (FS73R) or nonpersistent (FS73) MHV-68 infection. Mice were infected with the relevant virus, and then at various times postinfection, spleens cells were stained with major histocompatibility complex (MHC)/peptide tetramers and anti-CD8 antibody to measure the frequency of CD8 T cells recognizing the dominant epitope (17) (Fig. 1A and B). Consistent with our previous studies (16), the magnitude of the CD8 T cell response was greater in the FS73R-infected mice during the first 4 weeks of infection; however, memory populations were of similar size in both strains (Fig. 1A and B). Nrp1 expression was low in both cases during the early stages of infection (day 7 [d7]), but were significantly upregulated on d14, when CD8 T cell responses peak in MHV-68 infection (16, 17) (Fig. 1C). Nrp1 expression slowly declined after 14 days and had reduced to baseline expression levels by 60 days postinfection. While Nrp1 was induced with these kinetics in both FS73 and FS73R infections, the induction was significantly greater from days 14 to 21 after FS73 infection but not significantly different thereafter (Fig. 1C and D). This lead to the T cell response to FS73 being dominated by Nrp1 high expressing (Nrp1^hi^) cells during the acute infection (Fig. 1E), whereas there were more similar proportions of Nrp1^hi^ and Nrp1^lo^ cells at most times during the response to FS73R (Fig. 1F). In both cases, the majority of memory CD8 T cells at d100 were Nrp1^hi^ (Fig. 1E and F). These data indicate the absence of persistent infection leads to a greater induction of Nrp1 in the responding CD8 T cell population.

### Tamoxifen-induced Nrp1 excision and YFP expression in CD8 T cells.

**FIG 2:**
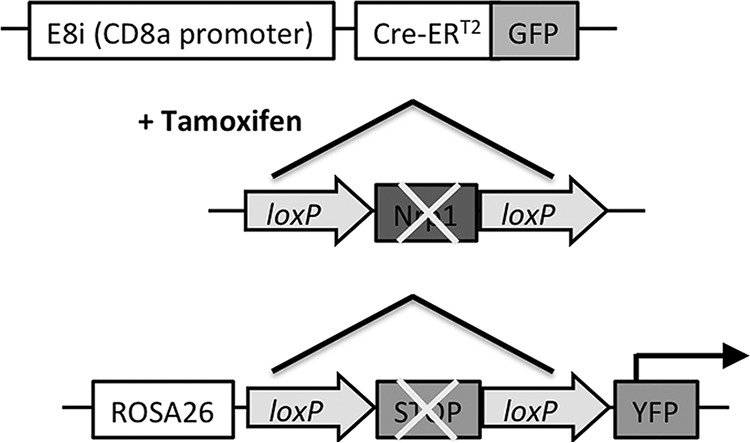
Schematic representation of Nrp1 E8i-CreERT2 R26-YFP mice. In the presence of tamoxifen, regions flanked by loxP sequences are excised, which deletes Nrp1 in CD8 T cells and removes the stop sequence downstream of the Rosa26 promoter, resulting in YFP expression in CD8 T cells.

**FIG 3:**
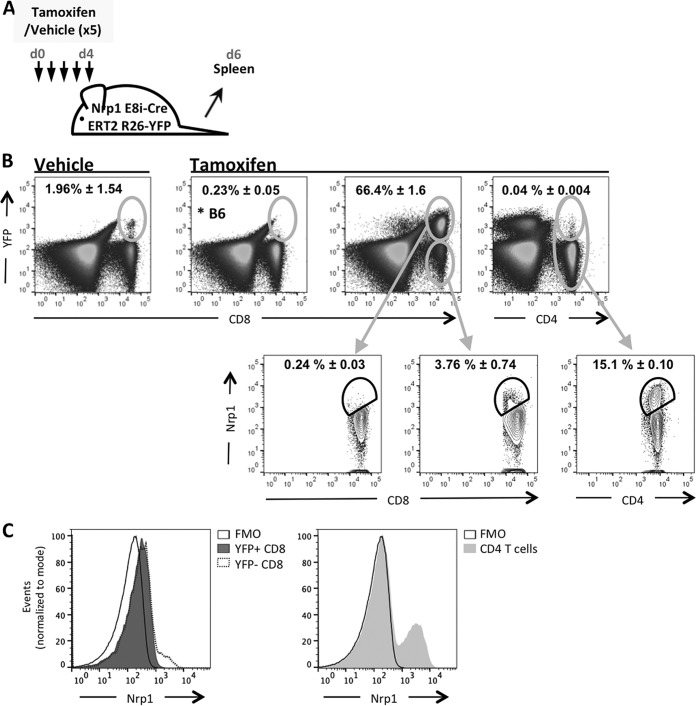
Tamoxifen administration effectively deletes Nrp1 from CD8 T cells in E8i-CreERT2 R26-YFP mice. (A) Protocol for tamoxifen treatment. (B) YFP expression was measured from CD8 or CD4 T cells after either tamoxifen or vehicle treatment in B6 or E8i-CreERT2 R26-YFP mice. Lower panels show Nrp1 staining on the indicated populations. (C) Histograms showing Nrp1 staining on CD8 (left) and CD4 (right) T cells from tamoxifen-treated E8i-CreERT2 R26-YFP mice. Representative data from at least two experiments are shown. Percentages shown are among the total CD8 or CD4 population, as appropriate.

To determine the role of Nrp1 in the antiviral CD8 T cell response, we wished to use an inducible knockout system that deletes Nrp1 selectively in CD8 T cells and only after induction by tamoxifen, as Nrp1 is important in the development of embryonic blood vessels and a systemic knockout is lethal (4). We therefore exploited conditional Nrp1 knockout transgenic mice (Nrp1 E8i-CreERT2 R26-YFP [Nrp1cKO]) (Fig. 2), where the E8i-CreERT2 cassette confers CD8 specificity (18) but Cre is translocated to the nucleus only after tamoxifen treatment. These mice also contain a floxed Nrp1 allele and a Rosa26-flox-stop-flox-yellow fluorescent protein (YFP) sequence resulting in deletion of Nrp1 and expression of YFP in cells where Cre is active. Then, we characterized these mice to verify inducible deletion by treating Nrp1cKO or C57BL/6NCrl (B6) mice with tamoxifen (1 mg/mouse) for 5 consecutive days (Fig. 3A) and measured Nrp1 expression on CD8 T cells 48 h after the last treatment. We observed that YFP expression was induced in CD8 T cells in Nrp1cKO mice (Fig. 3B, third panel) but not in B6 mice (Fig. 3B, second panel). While there was only a very small population of CD8 T cells expressing YFP after vehicle treatment (Fig. 3B, first panel), this rose to 66% following tamoxifen treatment (Fig. 3B, third panel). Tamoxifen-mediated induction of YFP was not observed in CD4 T cells in these mice (Fig. 3B, forth panel), confirming that Cre-mediated deletion was limited to CD8 T cells. To confirm YFP expression correlated with Nrp1 cell surface expression, we stained for Nrp1 on CD8 T cells from mice treated as described above and found this molecule was absent from the YFP^+^ population but present on a proportion of YFP^−^ cells (Fig. 3B, lower panels; Fig. 3C). After tamoxifen treatment, there was still an easily detectable population of Nrp1^+^ CD4 T cells (Fig. 3B and C), demonstrating the absence of Nrp1 deletion in this population. These data confirmed that tamoxifen treatment effectively abrogated Nrp1 expression in CD8 T cells and that cells lacking Nrp1 were marked by YFP fluorescence.

### Effect of Nrp1 deletion on CD8 T cell responses.

**FIG 4:**
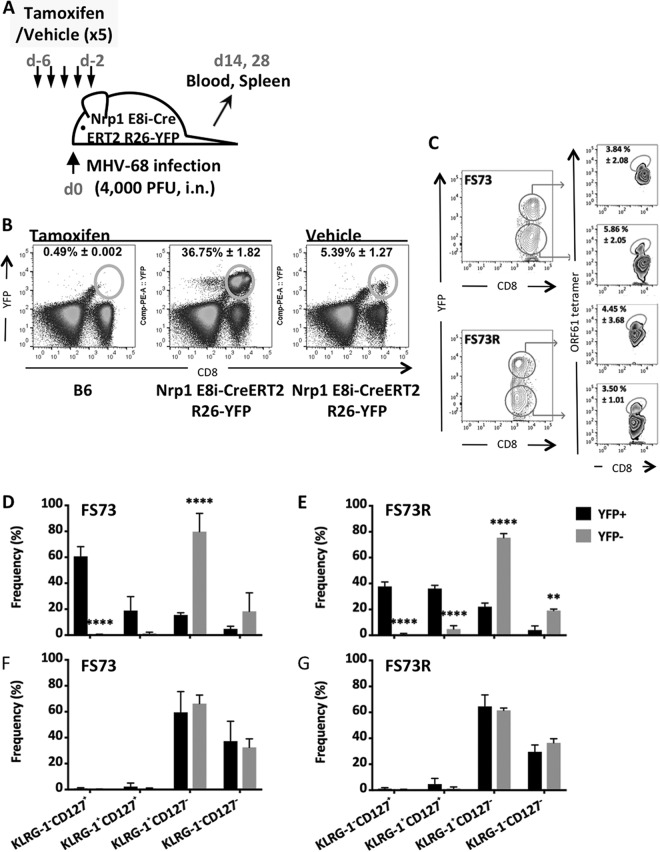
Effect of Nrp1 deletion on CD8 T cell responses. (A) Experimental design. (B) YFP expression in tamoxifen-treated B6 and tamoxifen- or vehicle-treated E8i-CreERT2 R26-YFP mice. (C) Evaluation of the size of the ORF61-specific population in the blood within either the YFP^+^ or YFP^−^ CD8 T cell populations in FS73 or FS73R-infected mice. (D and E) At d14 postinfection, blood was stained to identify CD8^+^ ORF61 tetramer^+^ cells, and the frequencies among this population are shown with respect to KLRG-1 and CD127 expression. (F and G) Spleen cells at d28 postinfection were stained as described for panels D and E. All data show means ± SDs from 4 to 5 mice per group. **, *P* < 0.01, ****, *P* < 0.0001. Representative data from at least two experiments.

To test the effect of Nrp1 on CD8 T cell differentiation during murine gammaherpesvirus infection, we treated Nrp1cKO mice with tamoxifen as described above and infected them with FS73 or FS73R 2 days after the last tamoxifen treatment (Fig. 4A). Vehicle-treated mice exhibited a very small YFP^+^ population, but this was greatly enlarged after tamoxifen treatment (Fig. 4B). To measure the effect of Nrp1 on CD8 T cell expansion and memory formation, we compared the proportions of YFP^+^ and YFP^−^ cells that stained with a tetramer identifying CD8 T cells recognizing the dominant ORF61 epitope. In this way, we had an internal control in each mouse, normalizing for variations in virus titers or other variables from mouse to mouse and enabling the use of paired statistical tests for significance. The frequencies of tetramer-positive cells were not significantly different in YFP^+^ and YFP^−^ cells at 14 days postinfection, regardless of virus strain (Fig. 4C), indicating the magnitude of the effector response was not altered by absence of Nrp1. However, we did detect differences in the differentiation status of the CD8 T cells. On day 14 in blood, we found that most tetramer^+^ YFP^+^ CD8 T cells (Nrp1 deleted) had the phenotype of precursors of memory cells (KLRG-1^−^ CD127^+^), whereas the majority of tetramer^+^ YFP^−^ CD8 T cells (expressing Nrp1) were terminally differentiated effector T cells (KLRG-1^+^ CD127^−^) (Fig. 4D and E). These results were comparable between persistent and nonpersistent infections, although with FS73R infection, the KLRG-1^+^ CD127^+^ population was as prominent as the KLRG-1^−^ CD127^+^ population (Fig. 4E). We previously showed that most memory CD8 T cells after infection with either the FS73 or FS73R virus do not upregulate CD127, unlike that seen in other infection models (16). Here, we observed most YFP^+^ and YFP^−^ CD8 T cells in the spleen became KLRG-1^+^ CD127^−^ by day 28 (Fig. 4F and G), indicating the absence of Nrp1 does not affect this phenotype. These data showed that Nrp1 favored the differentiation of effector CD8 T cells during the peak response and that in its absence, T cells differentiated preferentially toward the memory precursor phenotype. However, these changes did not endure to the memory phase, where Nrp1-sufficient and -deficient CD8 T cells had similar phenotypes.

### Effect of Nrp1 on the recall response.

**FIG 5:**
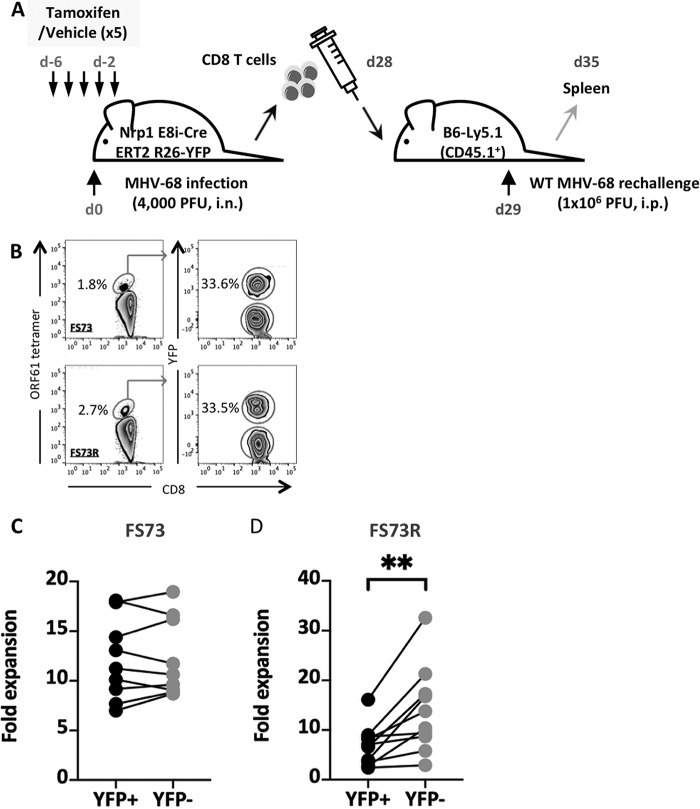
Effect on the recall response of Nrp1 deletion before infection. (A) Experimental design showing Nrp1 deletion before infection, and then adoptive transfer of memory CD8 T cells followed by infection of congenic secondary hosts. (B) Flow cytometry plots of adoptively transferred CD8 T cell populations. Plots show ORF61 tetramer^+^ CD8 T cell populations (left) and YFP^+^ populations within the tetramer^+^ population (right) from mice infected with FS73 (top) or FS73R (bottom). (C and D) Graphs showing expansion of YFP^+^ and YFP^−^ CD8^+^ ORF61 tetramer^+^ populations from mice infected originally with either FS73 (C) or FS73R (D) after secondary exposure to WT MHV-68. Joined lines represented paired samples (YFP^+^ and YFP^−^ cells from the same mouse). Data combined from 2 experiments are presented in panels C and D. **, *P* < 0.01.

Having observed a role for Nrp1 in the formation of memory CD8 T cells during the effector response, we next tested whether the quality of the resulting memory population was altered by rechallenge with virus. As in previous experiments, tamoxifen was administered before infection to delete Nrp1, and then splenic CD8 T cells were purified on d28 postinfection and adoptively transferred into congenic recipient mice (Fig. 5A). YFP expression was then used to identify the response from CD8 T cells with intact Nrp1 (YFP^−^) or deleted Nrp1 (YFP^+^) (Fig. 5B). Recipients were rechallenged with virus, and then 6 days later, spleens were removed and the expansion of adoptively transferred cells measured by flow cytometry. Memory CD8 T cells from mice infected with FS73 expanded comparably regardless of Nrp1 expression status (Fig. 5C), whereas the expansion of memory cells from FS73R donors was significantly reduced when they lacked Nrp1 (YFP^+^) (Fig. 5D).

**FIG 6:**
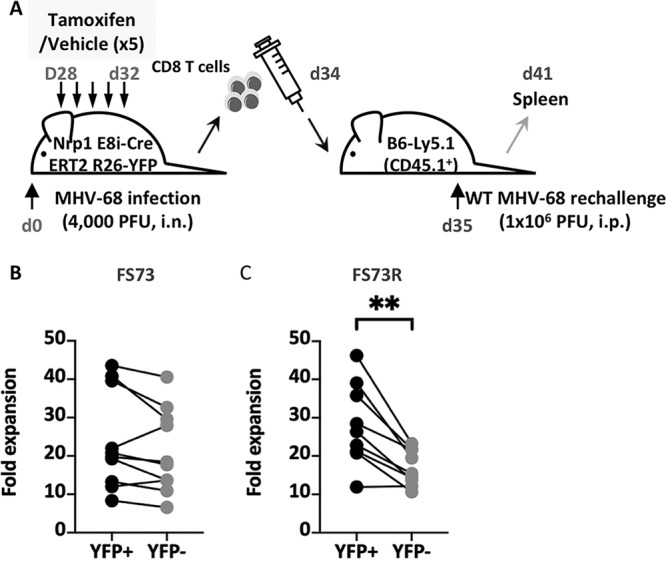
Effect of Nrp1 deletion just prior to the recall response. (A) Experimental design showing deletion of Nrp1 in memory cells, just before adoptive transfer to secondary congenic hosts. Graphs showing expansion of YFP^+^ and YFP^−^ CD8^+^ ORF61 tetramer^+^ populations from mice infected originally with either FS73 (B) or FS73R (C) after secondary exposure to WT MHV-68. Joined lines represented paired samples (YFP^+^ and YFP^−^ cells from the same mouse). Data combined from 2 experiments are presented. **, *P* < 0.01.

**FIG 7:**
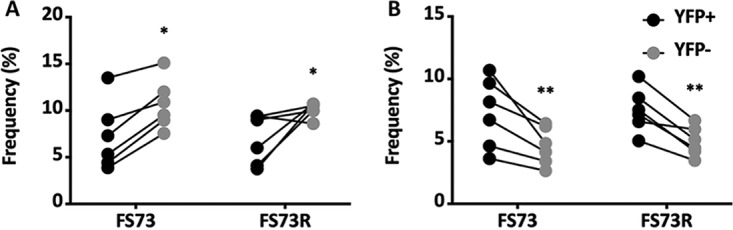
Cell viability and proliferation during recall responses where Nrp1 was deleted in memory cells. Experimental design was the same as that shown in Fig. 6A. (A) Graph of the percentages of dead cells after the recall response, determined by gating on CD8^+^ ORF61 tetramer^+^ cells and a LIVE/DEAD stain. (B) Mice were injected with 100 μl of 10 μM EdU intraperitoneally (i.p.) 16 h before euthanasia. EdU-incorporating proliferating cells were determined by gating on CD8^+^ ORF61 tetramer^+^ EdU^+^ cells after the recall response. All data show means ± SDs from 5 to 6 mice per group; *, *P* < 0.05, **, *P* < 0.01. Representative data from at least two experiments.

In the previous experiment, Nrp1 was absent both during the primary CD8 T cell response, where memory programming occurs, and also after CD8 T cells differentiated into memory cells. Next, we tested whether the absence of Nrp1 during the secondary response alone affected T cell expansion. To examine this, we performed a similar experiment, but this time, tamoxifen treatment started at d28 postinfection, and then spleens were harvested 2 days after the cessation of treatment (Fig. 6A). As before, purified CD8 T cells were transferred into congenic recipient mice that were then infected with MHV-68. Six days later, both YFP^+^ and YFP^−^ CD8 T cells from FS73-infected donors expanded to similar extents (Fig. 6B). However, YFP^+^ CD8 T cells from FS73R donor mice expanded to a greater extent than YFP^−^ cells (Fig. 6C), indicating Nrp1 expression during the secondary response limits the extent of T cell expansion. This greater expansion was attributed to better cell survival (lower frequencies of dead cells) (Fig. 7A) and more actively proliferating cells (5-ethynyl-2′-deoxyuridine [EdU] incorporation) (Fig. 7B) among the YFP^+^ population. These data indicated that Nrp1 restrains cellular proliferation and survival during the recall response, but it also plays a different role during the priming or postpriming phase of the response, promoting the development of memory cells capable of making an optimal recall response.

## DISCUSSION

This work shows clearly that neuropilin-1 plays important roles in both the differentiation of memory precursor cells and the capacity of memory precursor cells to mount a recall response. Cell surface Nrp1 expression was induced at the peak of the virus-specific CD8 T cell response and then declined slowly as these cells differentiated into memory cells. Interestingly, we detected a higher level of induction in mice infected with the nonpersistent FS73 strain. Our previous research showed a potent CD8 T cell response is induced after infection with either the FS73 or FS73R strain of MHV-68 (16). However, the capacity of the persistent strain to establish a latent infection in the spleen leads to splenomegaly, which increases the total number of virus-specific CD8 T cells and also likely creates a more proinflammatory environment in the spleen. While the regulation of Nrp1 in T cells is not known, it is possible Nrp1 expression is restrained by these proinflammatory signals. It is particularly interesting that Nrp1 deletion only affects recall responses in FS73R-infected mice, despite observing lower Nrp1 expression after infection with this persistent strain. The reasons for this effect are currently unclear but may be due to the fact that during persistent infection, there is sporadic reactivation, reexposing viral antigens to the T cell response. This would lead to antigen presentation by infected B cells, which express the Nrp1 ligand semaphorin 4A (8), potentially signaling to virus-specific memory CD8 T cells. In the acute infection with the nonpersistent FS73 strain, these interactions with semaphorin 4A-expressing antigen-presenting cells would be limited to lung-draining dendritic cells and infected lung epithelial cells but few B cells. Sporadic reexposure to viral antigen during persistent infection endows the memory CD8 T cells with the ability to elaborate antiviral effector functions more quickly (16), indicating a heightened state of readiness to counter the virus when it reactivates. Other evidence for these cells being in a different state of differentiation relative to that of memory cells in nonpersistently infected animals includes lower levels of Bcl-2, lower IL-2 production, and a faster turnover (16). Maintaining memory CD8 T cells in this differentiation state may be more dependent on Nrp1, which may also have an impact on their ability to mount a recall response.

Our data show that, regardless of virus strain, the absence of Nrp1 on CD8 T cells at the time of infection resulted in a bias toward KLRG-1^−^ CD127^+^ memory precursor phenotype cells at the expense of KLRG-1^+^ CD127^−^ effector cells at the peak of the response. However, this reduction in the proportion of effector cells did not reduce the overall magnitude of the response, indicating T cell proliferation is unaffected. Nrp1 therefore may have a role in restraining the differentiation of memory precursors while favoring the differentiation of effector T cells. This is counter to what may be expected based on a previous report that found Nrp1 at the immunological synapse acts through the phosphatase PTEN to restrain Akt phosphorylation in Tregs (8). In CD8 T cells, Akt promotes effector cell differentiation and glycolytic metabolism acting through mTOR and T-bet (19), whereas inhibition of Akt promotes memory differentiation (20, 21). Therefore, Nrp1 may be expected to restrict Akt phosphorylation and promote memory differentiation, counter to what we observed. This is likely due to differences in the signal transduction pathways present in Tregs and CD8 T cells. Further work is necessary to uncover the underlying mechanism and to determine why this apparent skewing to memory precursors does not result in a larger long-term memory population.

Our use of a conditional deletion model where Nrp1 expression is abrogated on approximately half the CD8 T cells, and the other half retain Nrp1 expression, allowed us to perform very precise internally controlled experiments measuring the secondary CD8 T cell response. A complex picture of the roles of Nrp1 during the recall response emerges, with contrasting functions during different stages of the response. When deleted prior to infection, the absence of Nrp1 reduced the size of the recall response, indicating Nrp1 promotes the ability of memory cells to expand upon antigen reexposure. While Nrp1 expression was most prominent on effector cells, it was still expressed at a low level on memory CD8 T cells at d28 postinfection. Therefore, Nrp1 could be acting in this context by programming appropriate differentiation of memory cells during the effector response, signaling during the contraction and early memory phases, or potentially both.

To interrogate the function of Nrp1 during the recall response itself, we allowed the effector and memory response to develop in the presence of Nrp1 and then deleted it just prior to memory cell harvest and cell transfer to secondary recipients. In this context, deletion of Nrp1 enhanced the recall response by promoting T cell viability and proliferation. Although we cannot rule it out, we consider it unlikely the protocol we used to delete Nrp1 in memory CD8 T cells, terminating 2 days prior to transfer (Fig. 6A), affected the differentiation state of the CD8 T cells in a manner similar to deletion prior to infection. It seems more likely Nrp1 pays a direct role during the recall response, serving to repress CD8 T cell proliferation and limit cell survival, presumably to regulate the size of the T cell response and prevent immunopathology.

A previous report detailed a role for Nrp1 in the initiation of a primary T cell response from human T cells (22). Antibody blockade of Nrp1 reduced the proliferation of naive T cells 50% to 60% when stimulated with allogeneic dendritic cells, and Nrp1 was shown to cluster to dendritic cell-T cell contact areas. This contrasts with our finding that the primary response was not affected by the presence or absence of Nrp1. There are many differences between this report and our study, including the use of human cells versus mice, studying whole T cell populations versus CD8 T cells, and studying allogeneic responses versus antigen-specific responses. One additional potential reason for the difference in our findings is that antibody blockade of Nrp1 at the cellular interface may result in steric hindrance of other important interactions, thereby reducing signaling through the immunologic synapse. This is not a concern in our studies, as we used genetic deletion to ablate Nrp1 expression.

Our finding that Nrp1 expressed during the secondary response represses the proliferative response can be seen as consistent with previous studies reporting inhibition of T cell responses by Nrp1. Semaphorin 3A (Sema3A), a physiological ligand of Nrp1 (23–25), inhibits *in vitro* dendritic cell (DC)-T cell interactions (26) and tumor-T cell interactions (27), and by blocking Sema3A, hence suppressing the downstream signaling involving Nrp1, T cell activation and proliferation were restored. However, these studies used whole T cell populations, which include Tregs, and so the inhibition observed may have been due to Treg-mediated suppression, in which Nrp1 plays a key role (8, 28, 29), rather than direct interactions with CD8 T cells.

A previous report identified Nrp1 upregulation on anergic mouse CD8 T cells, but Nrp1 did not appear to play any role in the tolerant phenotype (30). However, to our knowledge, our study is the first to interrogate Nrp1 function directly on an antigen-specific antiviral CD8 T cell response *in vivo*. It highlights complex roles for this molecule in memory CD8 T cell differentiation and the secondary immune response, which depend upon the stage of the response and the nature of the infection. While Nrp1-semaphorin interactions are known to have an important impact on antitumor immunity, this study shows additional roles in antiviral immunity and the memory CD8 T cell response.

## MATERIALS AND METHODS

### Mice and MHV-68 infection.

C57BL/6NCrl (B6) mice were originally obtained from Charles River Laboratory. Nrp1 E8i-CreERT2 R26-YFP mice were kindly provided by Dario A. Vignali from The University of Pittsburgh. The primers used to genotype the strain were as shown in Table 1. Mice were maintained under specific pathogen-free conditions in the Dartmouth Center for Comparative Medicine and Research. The Animal Care and Use Committee of Dartmouth College approved all animal experiments. MHV-68 containing a frameshift mutation in ORF73 (FS73) and the revertant virus (FS73R) were originally obtained from Stacey Efstathiou at The University of Cambridge, United Kingdom. Seven-week-old mice were primarily infected with 4 × 10^3^ PFU by the intranasal route in 30 μl Hanks’ balanced salt solution (HBSS) and rechallenged with 1 × 10^6^ PFU wild-type (WT) MHV-68 by the intraperitoneal route.

**TABLE 1:** Primers used to screen transgenic mice used in these studies

Primer name	Sequence
Nrp1-Forward	AGGTTAGGCTTCAGGCCAAT
Nrp1-Reverse	GCAGATCTCTTCCCTGCAAC
Rosa26-YFP-1	GCGAAGAGTTTGTCCTCAACC
Rosa26-YFP-2	GGAGCGGGAGAAATGGATATG
Rosa26-YFP-3	AAAGTCGCTCTGAGTTGTTAT
E8i-Cre-IC-1	CTAGGCCACAGAATTGAAAGATCT
E8i-Cre-IC-2	GTAGGTGGAAATTCTAGCATCATCC
E8i-Cre-ER7-For	CCACCGAGTCCTGGACAAGATCAC
E8i-Cre-IRES-Rev	CCTCGACTAAACACATGTAAAGCATG

### Tamoxifen treatment, T cell purification, and adoptive transfer.

Tamoxifen (VWR) was suspended in 5% (vol/vol) ethanol (EtOH)-corn oil (Ward’s Science), warmed at 37°C for at least 30 min before treatment, and 1 mg/100 μl/mouse was given intraperitoneally for 5 consecutive days, starting at day −6 relative to infection (d−6) for early Nrp1 deletion and d28 for late Nrp1 deletion. CD8 T cells were purified from the spleens of early Nrp1-deleted (d28) or late Nrp1-deleted (d34) Nrp1 E8i-CreERT2 R26-mice using EasySep Mouse CD8 T cell isolation Kits (Stemcell Technologies) according to the manufacturer’s instructions. Single T cell preparations were >95% pure as determined by flow cytometry. CD8 T cell populations containing 2 × 10^4^ ORF61 tetramer^+^ memory CD8 T cells were injected by the retro-orbital route into B6-Ly5.1 recipients. The recipients were rechallenged 1 day after the adoptive T cell transfer, and splenocytes were collected on day 6 postinfection.

### Cell preparation, flow cytometry, and proliferation assay.

Single-cell suspensions from spleen were prepared by passing them through cell strainers and were resuspended in Gey’s solution (150 mM NH_4_Cl, 10 mM KHCO_3_, and 0.05% phenol red) for 5 min to lyse red cells. Cell suspensions were then filtered through a 70-μm nylon cell strainer (BD Biosciences), washed, and resuspended in phosphate-buffered saline (PBS) with 2% bovine growth serum (BGS) and allophycocyanin (APC)-conjugated tetramer specific for the MHV-68 dominant epitope (ORF61; NIH tetramer core facility) at room temperature for 1 h, followed by 10 μg/ml Fc Block (2.4G2; Dartlab) on ice for 10 min before staining with the following fluorochrome-conjugated antibodies (Abs): anti-cluster of differentiation (CD) 8-BV510 (CD8; 53-6.7), anti-CD45.2-BV421 (104), anti-CD45.2-BV650 (104), anti-CD304-BV421 (neuropilin-1; 3E12), and anti-KLRG-1-phycoerythrin (PE)-Cy7 (2F1/KLRG-1; all from BioLegend), as well as anti-CD4-APC (GK.15) and anti-CD127-APC 780 (A7R34; all from eBioscience). The LIVE/DEAD Fixable Near-IR dead cell stain kit (Thermo Fisher) was used to assess cell viability, and a Click-iT Plus EdU Pacific Blue flow cytometry assay kit (Thermo Fisher) was used to assess cell proliferation. Cells were analyzed with MACSQuant (Miltenyi) FACS Aria (Becton, Dickinson) or CytoFLEX S (Beckman Coulter) flow cytometers at the Dartlab flow cytometry core facility.

### Statistical analysis.

Two-way analysis of variance (ANOVA) with Sidak’s or Dunnett’s multiple-comparison test was used (GraphPad Prism version 7.0). *P* values of less than 0.05 were considered statistically significant.

## References

[1] Plein A, Fantin A, Ruhrberg C 2014 Neuropilin regulation of angiogenesis, arteriogenesis, and vascular permeability. Microcirculation 21:315–323. doi:10.1111/micc.12124. 24521511 PMC4230468

[2] Kolodkin AL, Matthes DJ, Goodman CS 1993 The semaphorin genes encode a family of transmembrane and secreted growth cone guidance molecules. Cell 75:1389–1399. doi:10.1016/0092-8674(93)90625-Z. 8269517

[3] Soker S, Takashima S, Miao HQ, Neufeld G, Klagsbrun M 1998 Neuropilin-1 is expressed by endothelial and tumor cells as an isoform-specific receptor for vascular endothelial growth factor. Cell 92:735–745. doi:10.1016/S0092-8674(00)81402-6. 9529250

[4] Kawasaki T, Kitsukawa T, Bekku Y, Matsuda Y, Sanbo M, Yagi T, Fujisawa H 1999 A requirement for neuropilin-1 in embryonic vessel formation. Development 126:4895–4902. 10518505 10.1242/dev.126.21.4895

[5] Xin Y, Li J, Wu J, Kinard R, Weekes CD, Patnaik A, LoRusso P, Brachmann R, Tong RK, Yan Y, Watts R, Bai S, Hegde PS 2012 Pharmacokinetic and pharmacodynamic analysis of circulating biomarkers of anti-NRP1, a novel antiangiogenesis agent, in two phase I trials in patients with advanced solid tumors. Clin Cancer Res 18:6040–6048. doi:10.1158/1078-0432.CCR-12-1652. 22962439

[6] Pan Q, Chanthery Y, Liang W-C, Stawicki S, Mak J, Rathore N, Tong RK, Kowalski J, Yee SF, Pacheco G, Ross S, Cheng Z, Le Couter J, Plowman G, Peale F, Koch AW, Wu Y, Bagri A, Tessier-Lavigne M, Watts RJ 2007 Blocking neuropilin-1 function has an additive effect with anti-VEGF to inhibit tumor growth. Cancer Cell 11:53–67. doi:10.1016/j.ccr.2006.10.018. 17222790

[7] Barr MP, Byrne AM, Duffy AM, Condron CM, Devocelle M, Harriott P, Bouchier-Hayes DJ, Harmey JH 2005 A peptide corresponding to the neuropilin-1-binding site on VEGF_165_ induces apoptosis of neuropilin-1-expressing breast tumour cells. Br J Cancer 92:328–333. doi:10.1038/sj.bjc.6602308. 15655556 PMC2361857

[8] Delgoffe GM, Woo S-R, Turnis ME, Gravano DM, Guy C, Overacre AE, Bettini ML, Vogel P, Finkelstein D, Bonnevier J, Workman CJ, Vignali DAA 2013 Stability and function of regulatory T cells is maintained by a neuropilin-1-semaphorin-4a axis. Nature 501:252–256. doi:10.1038/nature12428. 23913274 PMC3867145

[9] Yadav M, Louvet C, Davini D, Gardner JM, Martinez-Llordella M, Bailey-Bucktrout S, Anthony BA, Sverdrup FM, Head R, Kuster DJ, Ruminski P, Weiss D, Von Schack D, Bluestone JA 2012 Neuropilin-1 distinguishes natural and inducible regulatory T cells among regulatory T cell subsets *in vivo*. J Exp Med 209:1713–1722. doi:10.1084/jem.20120822. 22966003 PMC3457729

[10] Weiss JM, Bilate AM, Gobert M, Ding Y, Curotto de Lafaille MA, Parkhurst CN, Xiong H, Dolpady J, Frey AB, Ruocco MG, Yang Y, Floess S, Huehn J, Oh S, Li MO, Niec RE, Rudensky AY, Dustin ML, Littman DR, Lafaille JJ 2012 Neuropilin 1 is expressed on thymus-derived natural regulatory T cells, but not mucosa-generated induced Foxp3^+^ T reg cells. J Exp Med 209:1723–1742. doi:10.1084/jem.20120914. 22966001 PMC3457733

[11] Yuan Q, Hong S, Shi B, Kers J, Li Z, Pei X, Xu L, Wei X, Cai M 2013 CD4^+^CD25^−^Nrp1^+^ T cells synergize with rapamycin to prevent murine cardiac allorejection in immunocompetent recipients. PLoS One 8:e61151. doi:10.1371/journal.pone.0061151. 23577203 PMC3618334

[12] Shikhagaie MM, Björklund ÅK, Mjösberg J, Erjefält JS, Cornelissen AS, Ros XR, Bal SM, Koning JJ, Mebius RE, Mori M, Bruchard M, Blom B, Spits H 2017 Neuropilin-1 is expressed on lymphoid tissue residing LTi-like group 3 innate lymphoid cells and associated with ectopic lymphoid aggregates. Cell Rep 18:1761–1773. doi:10.1016/j.celrep.2017.01.063. 28199847 PMC5318658

[13] Kaech SM, Tan JT, Wherry EJ, Konieczny BT, Surh CD, Ahmed R 2003 Selective expression of the interleukin 7 receptor identifies effector CD8 T cells that give rise to long-lived memory cells. Nat Immunol 4:1191–1198. doi:10.1038/ni1009. 14625547

[14] Fuse S, Molloy MJ, Usherwood EJ 2008 Immune responses against persistent viral infections: possible avenues for immunotherapeutic interventions. Crit Rev Immunol 28:159–183. doi:10.1615/CritRevImmunol.v28.i2.40. 18540829

[15] Fowler P, Marques S, Simas JP, Efstathiou S 2003 ORF73 of murine herpesvirus-68 is critical for the establishment and maintenance of latency. J Gen Virol 84:3405–3416. doi:10.1099/vir.0.19594-0. 14645921

[16] Obar JJ, Fuse S, Leung EK, Bellfy SC, Usherwood EJ 2006 Gammaherpesvirus persistence alters key CD8 T-cell memory characteristics and enhances antiviral protection. J Virol 80:8303–8315. doi:10.1128/JVI.00237-06. 16912282 PMC1563881

[17] Stevenson PG, Belz GT, Altman JD, Doherty PC 1999 Changing patterns of dominance in the CD8^+^ T cell response during acute and persistent murine gamma-herpesvirus infection. Eur J Immunol 29:1059–1067. doi:10.1002/(SICI)1521-4141(199904)29:04<1059::AID-IMMU1059>3.0.CO;2-L. 10229071

[18] Maekawa Y, Minato Y, Ishifune C, Kurihara T, Kitamura A, Kojima H, Yagita H, Sakata-Yanagimoto M, Saito T, Taniuchi I, Chiba S, Sone S, Yasutomo K 2008 Notch2 integrates signaling by the transcription factors RBP-J and CREB1 to promote T cell cytotoxicity. Nat Immunol 9:1140–1147. doi:10.1038/ni.1649. 18724371

[19] Araki K, Turner AP, Shaffer VO, Gangappa S, Keller SA, Bachmann MF, Larsen CP, Ahmed R 2009 mTOR regulates memory CD8 T-cell differentiation. Nature 460:108–112. doi:10.1038/nature08155. 19543266 PMC2710807

[20] Kim EH, Sullivan JA, Plisch EH, Tejera MM, Jatzek A, Choi KY, Suresh M 2012 Signal integration by Akt regulates CD8 T cell effector and memory differentiation. J Immunol 188:4305–4314. doi:10.4049/jimmunol.1103568. 22467649 PMC3331885

[21] Hand TW, Cui W, Jung YW, Sefik E, Joshi NS, Chandele A, Liu Y, Kaech SM 2010 Differential effects of STAT5 and PI3K/AKT signaling on effector and memory CD8 T-cell survival. Proc Natl Acad Sci U S A 107:16601–16606. doi:10.1073/pnas.1003457107. 20823247 PMC2944719

[22] Tordjman R, Lepelletier Y, Lemarchandel V, Cambot M, Gaulard P, Hermine O, Roméo P-H 2002 A neuronal receptor, neuropilin-1, is essential for the initiation of the primary immune response. Nat Immunol 3:477–482. doi:10.1038/ni789. 11953749

[23] Kolodkin AL, Levengood DV, Rowe EG, Tai YT, Giger RJ, Ginty DD 1997 Neuropilin is a semaphorin III receptor. Cell 90:753–762. doi:10.1016/S0092-8674(00)80535-8. 9288754

[24] Takahashi T, Fournier A, Nakamura F, Wang LH, Murakami Y, Kalb RG, Fujisawa H, Strittmatter SM 1999 Plexin-neuropilin-1 complexes form functional semaphorin-3A receptors. Cell 99:59–69. doi:10.1016/S0092-8674(00)80062-8. 10520994

[25] Gu C, Limberg BJ, Whitaker GB, Perman B, Leahy DJ, Rosenbaum JS, Ginty DD, Kolodkin AL 2002 Characterization of neuropilin-1 structural features that confer binding to semaphorin 3A and vascular endothelial growth factor 165. J Biol Chem 277:18069–18076. doi:10.1074/jbc.M201681200. 11886873

[26] Lepelletier Y, Moura IC, Hadj-Slimane R, Renand A, Fiorentino S, Baude C, Shirvan A, Barzilai A, Hermine O 2006 Immunosuppressive role of semaphorin-3A on T cell proliferation is mediated by inhibition of actin cytoskeleton reorganization. Eur J Immunol 36:1782–1793. doi:10.1002/eji.200535601. 16791896

[27] Catalano A, Caprari P, Moretti S, Faronato M, Tamagnone L, Procopio A 2006 Semaphorin-3A is expressed by tumor cells and alters T-cell signal transduction and function. Blood 107:3321–3329. doi:10.1182/blood-2005-06-2445. 16380453

[28] Bruder D, Probst-Kepper M, Westendorf AM, Geffers R, Beissert S, Loser K, von Boehmer H, Buer J, Hansen W 2004 Frontline: Neuropilin-1: a surface marker of regulatory T cells. Eur J Immunol 34:623–630. doi:10.1002/eji.200324799. 14991591

[29] Hansen W, Hutzler M, Abel S, Alter C, Stockmann C, Kliche S, Albert J, Sparwasser T, Sakaguchi S, Westendorf AM, Schadendorf D, Buer J, Helfrich I 2012 Neuropilin 1 deficiency on CD4^+^ Foxp3^+^ regulatory T cells impairs mouse melanoma growth. J Exp Med 209:2001–2016. doi:10.1084/jem.20111497. 23045606 PMC3478934

[30] Jackson SR, Berrien-Elliott M, Yuan J, Hsueh EC, Teague RM 2014 Neuropilin-1 expression is induced on tolerant self-reactive CD8^+^ T cells but is dispensable for the tolerant phenotype. PLoS One 9:e110707. doi:10.1371/journal.pone.0110707. 25343644 PMC4208794

